# Audit of shared-care lithium monitoring in a large rural GP practice

**DOI:** 10.1192/bjo.2021.834

**Published:** 2021-06-18

**Authors:** Thomas Cranshaw

**Affiliations:** Cumbria, Northumberland Tyne and Wear Foundation Trust

## Abstract

**Aims:**

To compare monitoring of lithium treatment with shared care lithium monitoring agreements in a large rural GP practice.

**Background:**

A ‘near miss’ event with a patient with drug induced long QT syndrome highlighted a need for an audit of lithium monitoring at a large rural GP practice.

The practice had entered into shared-care monitoring agreements with the local mental health care trust. Under these agreements, responsibility for physical monitoring of lithium treatment was assumed by the practice.

**Method:**

Using audit functions built into the IT system, all patients at the practice who were currently prescribed lithium-containing medications were identified (n = 28). Individual monitoring standards were determined for each patient based on the shared care agreement. These varied depending on age and comorbidity. Monitoring data obtained from medical records was compared against the individualised monitoring requirement.

**Result:**

The key finding was that 26% of patients for whom annual ECGs were indicated according to the shared care agreement had received an ECG in the past year. 78% of patients had a lithium level recorded in the previous 3 months. 81% of patients had a renal function test within their monitoring requirements. 52% of patients had lipid measurement in the previous year.

**Conclusion:**

There is a great degree of heterogeneity in the extent to which shared care monitoring agreements are achieved. It is noted that those standards to which a Quality Outcome Framework incentive applied had a greater chance of being met. Worryingly, the QOF statements relating to lithium treatment have now been retired as of April 2019. It is suggested that psychiatrists are aware of the challenges primary care faces when monitoring lithium treatment.

